# RBBP6 activates the pre-mRNA 3′ end processing machinery in humans

**DOI:** 10.1101/gad.349223.121

**Published:** 2022-02-01

**Authors:** Vytaute Boreikaite, Thomas S. Elliott, Jason W. Chin, Lori A. Passmore

**Affiliations:** Medical Research Council Laboratory of Molecular Biology, Cambridge CB2 0QH, United Kingdom

**Keywords:** RNA, endonuclease, gene expression, polyadenylation

## Abstract

3′ end processing of most human mRNAs is carried out by the cleavage and polyadenylation specificity factor (CPSF; CPF in yeast). Endonucleolytic cleavage of the nascent pre-mRNA defines the 3′ end of the mature transcript, which is important for mRNA localization, translation, and stability. Cleavage must therefore be tightly regulated. Here, we reconstituted specific and efficient 3′ endonuclease activity of human CPSF with purified proteins. This required the seven-subunit CPSF as well as three additional protein factors: cleavage stimulatory factor (CStF), cleavage factor IIm (CFIIm), and, importantly, the multidomain protein RBBP6. Unlike its yeast homolog Mpe1, which is a stable subunit of CPF, RBBP6 does not copurify with CPSF and is recruited in an RNA-dependent manner. Sequence and mutational analyses suggest that RBBP6 interacts with the WDR33 and CPSF73 subunits of CPSF. Thus, it is likely that the role of RBBP6 is conserved from yeast to humans. Overall, our data are consistent with CPSF endonuclease activation and site-specific pre-mRNA cleavage being highly controlled to maintain fidelity in mRNA processing.

## Abstract (precis)

In this study, Boreikaite et al. reconstituted specific and efficient 3′ endonuclease activity of human CPSF with purified proteins. This required the seven-subunit CPSF as well as three additional protein factors: cleavage stimulatory factor (CStF), cleavage factor IIm (CFIIm), and, importantly, the multidomain protein RBBP6.

Eukaryotic protein-coding pre-mRNAs undergo multiple processing steps during transcription by RNA polymerase II. These include 5′ capping, splicing, and 3′ end processing (Hocine et al. 2010). During this latter process, a cleavage event defines the 3′ end of the mature mRNA and is linked to transcription termination (Buratowski 2005; Liu and Moore 2021). A poly(A) tail is added to the resultant free 3′ end, marking the mRNA for nuclear export and controlling mRNA stability and translational efficiency in the cytoplasm (Passmore and Coller 2022). Thus, 3′ cleavage and polyadenylation are critical to the production of functional protein-coding transcripts.

In humans, cleavage and polyadenylation are carried out by a seven-subunit protein complex known as cleavage and polyadenylation specificity factor (CPSF) (Kumar et al. 2019; Zhang et al. 2020). CPSF is comprised of two stable subcomplexes: mammalian polyadenylation specificity factor (mPSF) and mammalian cleavage factor (mCF). These are equivalent to the polymerase module and nuclease module, respectively, of the yeast cleavage and polyadenylation factor (CPF) (Casañal et al. 2017). mPSF contains four subunits: CPSF160, WDR33, CPSF30, and hFip1 (Schönemann et al. 2014). Structures of mPSF/polymerase module in apo and RNA-bound states have recently been elucidated (Casañal et al. 2017; Clerici et al. 2017, 2018; Sun et al. 2018). These showed how the CPSF30 and WDR33 subunits recognize the hexameric polyadenylation signal (PAS) sequence, most commonly AAUAAA, thereby recruiting CPSF to cleavage sites on pre-mRNAs. The poly(A) polymerase enzyme (PAP) is not a stable subunit of human CPSF but is instead recruited to cleaved transcripts by hFip1 (Kaufmann et al. 2004; Chan et al. 2014). mCF consists of three subunits: CPSF73, CPSF100, and symplekin (Zhang et al. 2020). CPSF73 is a zinc-dependent RNA endonuclease that belongs to the metallo-β-lactamase family. CPSF100 is a pseudonuclease that is structurally homologous to CPSF73 (Mandel et al. 2006). mCF is tethered to mPSF through a conserved interaction between CPSF160 and a peptide within CPSF100, known as the mPSF interaction motif (PIM) (Zhang et al. 2020; Rodríguez-Molina et al. 2021).

To ensure that mature transcripts of a correct length are produced, pre-mRNAs must be cleaved at specific sites. Deregulation of this process can result in transcriptional defects and nonfunctional transcripts, and can lead to human disease (Curinha et al. 2014). In vitro, purified CPSF/CPF is an inherently inactive endonuclease, which presumably must be activated by accessory factors to enable strict regulation of 3′ cleavage (Mandel et al. 2006; Hill et al. 2019; Zhang et al. 2020). For example, cleavage stimulatory factor (CStF) and cleavage factor IIm (CFIIm) are both multisubunit protein complexes implicated in cleavage (Takagaki et al. 1990; De Vries et al. 2000). CStF has been shown to bind a G/U-rich region downstream from the cleavage site on pre-mRNAs and to provide specificity for poly(A) site selection (Takagaki and Manley 1997). Another accessory factor, cleavage factor Im (CFIm), is not essential for 3′ cleavage but recruits CPSF to pre-mRNAs containing an upstream UGUA motif and contributes to the use of alternative polyadenylation sites in human cells (Zhu et al. 2018).

The cleavage activity of human CPSF has been studied by functional genomics and by in vitro experiments in fractionated nuclear extracts prepared from cultured human cells (for recent examples, see Eaton et al. 2018; Schäfer et al. 2018). However, the full protein composition of partially purified 3′ end processing machinery from nuclear extract is not known, making it difficult to infer molecular mechanisms. Moreover, generating mutants of endogenous proteins to test hypotheses is cumbersome.

To enable detailed mechanistic studies of CPSF endonuclease activation, an in vitro assay containing a well-defined set of highly pure proteins is required. Recently, this has been achieved for the human histone pre-mRNA 3′ end processing complex, which shares the endonuclease subunit CPSF73 but differs from CPSF in most of its other subunits and its mechanism of RNA recognition (Sun et al. 2020; Gutierrez et al. 2021). The endonuclease activity of the budding yeast CPF complex has also been reconstituted from purified recombinant proteins (Hill et al. 2019). The minimal active subcomplex in yeast, called core CPF, contains orthologs of CPSF subunits as well as an additional protein: Mpe1. We recently showed that Mpe1 is an essential activator of the CPF endonuclease (Rodríguez-Molina et al. 2021). However, while many aspects of 3′ end processing are conserved, there appear to be some differences between the yeast and human machineries, including in RNA specificity and recognition (Tian and Graber 2012; Rodríguez-Molina et al. 2021). The human ortholog of Mpe1, RBBP6, has been implicated in pre-mRNA 3′ end processing in humans (Shi et al. 2009; Di Giammartino et al. 2014). Whether it plays a direct role in the cleavage reaction remains unclear.

Here, we reconstituted CPSF from purified proteins that is active in both cleavage and polyadenylation. We demonstrate that human RBBP6 is required for the activation of 3′ end cleavage even though it is not a stable subunit of CPSF. Our results show that the mechanism of endonuclease activation by Mpe1/RBBP6 is likely to be highly conserved.

## Results

### CStF, CFIIm, and RBBP6 are required for activation of CPSF endonuclease

**Figure 1.**
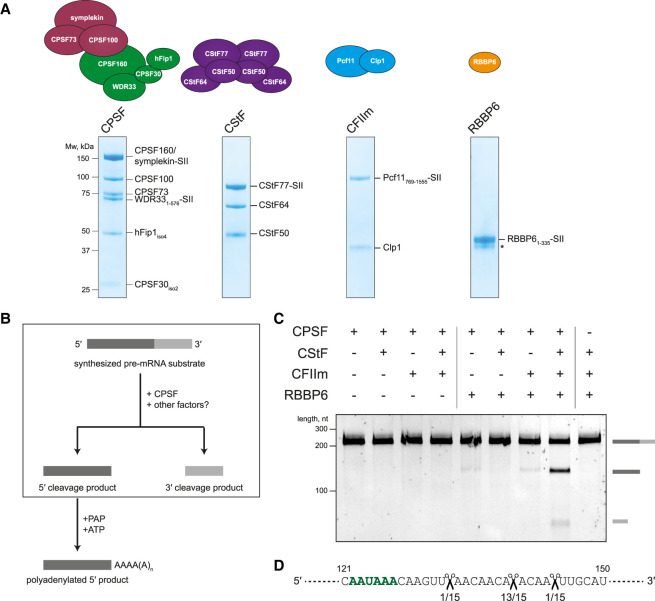
CStF, CFIIm, and RBBP6 are required for activation of the CPSF endonuclease. (*A*) Schematic representations and SDS-PAGE analyses of the purified proteins used in the in vitro endonuclease assays. Residue boundaries and alternative isoforms are indicated for truncated proteins. An asterisk denotes degradation products. (SII) StrepII tag. (*B*) Schematic representation of the in vitro pre-mRNA 3′ end processing assay. The cleavage reaction is boxed out. The polyadenylation step was not assayed here. (*C*) Denaturing gel electrophoresis of the SV40 pre-mRNA substrate after incubation with various combinations of human 3′ end processing factors. The full-length and cleaved RNAs are shown schematically at the *right*. (*D*) Part of the sequence of the SV40 pre-mRNA substrate with the experimentally determined CPSF cleavage sites indicated (scissors). The frequency of a particular cleavage site identified by sequencing of 15 cleavage products is shown *below*. The polyadenylation signal (PAS) sequence is marked in green.

To gain insight into how the human CPSF endonuclease is activated, we attempted to reconstitute pre-mRNA cleavage activity from purified recombinant proteins. We used baculovirus-mediated expression in insect cells to produce highly pure protein complexes predicted to be directly involved in canonical pre-mRNA 3′ end processing. This included CPSF (assembled from individually purified mPSF and mCF subcomplexes) as well as the accessory factors CStF and CFIIm (Fig. 1A). We used short isoforms of CPSF30 and hFip1, and also removed unstructured regions from WDR33 and the CFIIm subunit Pcf11 to facilitate purification (Schäfer et al. 2018; Sun et al. 2018). We hypothesized that the conserved region of the multidomain protein RBBP6 (residues 1–335) might also be required for endonuclease activation. RBBP6 did not copurify with CPSF and was therefore expressed and purified separately. As a model pre-mRNA substrate, we used a 218-nt fragment of the SV40 pre-mRNA, which has been shown to be cleaved efficiently in vivo (Ryner and Manley 1987; Kwon et al. 2021). We omitted the PAP enzyme and ATP from the reactions to focus on the cleavage step of pre-mRNA 3′ end processing (Fig. 1B).

We tested various combinations of 3′ end processing factors in cleavage assays and analyzed the results by denaturing gel electrophoresis of RNA (Fig. 1C). No cleavage activity was observed when the SV40 pre-mRNA was incubated with CPSF alone. Addition of CStF and CFIIm, either individually or together, failed to activate CPSF. However, addition of RBBP6 activated CPSF in the presence of CStF and CFIIm, promoting efficient cleavage of the pre-mRNA substrate. Previous assays in nuclear extract used molecular crowding agents such as polyvinyl alcohol (Adamson et al. 2005), but these were not required here. Omitting CPSF from the reaction did not lead to substrate cleavage, showing that the observed endonuclease activity cannot be attributed to potential contaminants that copurify with the accessory proteins. Overall, we determined that activation of the CPSF endonuclease requires three additional protein factors: CStF, CFIIm, and RBBP6.

To identify the precise CPSF cleavage site on the SV40 pre-mRNA substrate, we sequenced several 5′ cleavage products. This revealed that the majority (13 out of 15) of cleaved RNAs were cut 13 nt downstream from the PAS within a CA|A motif, where | indicates the cleavage site (Fig. 1D; Sheets et al. 1987). This is consistent with the known sequence preference of 3′ endonucleases and with previous observations that pre-mRNAs in cells are cleaved 10–30 nt downstream from the PAS (Beaudoing et al. 2000; Hill et al. 2019).

**Figure 2.**
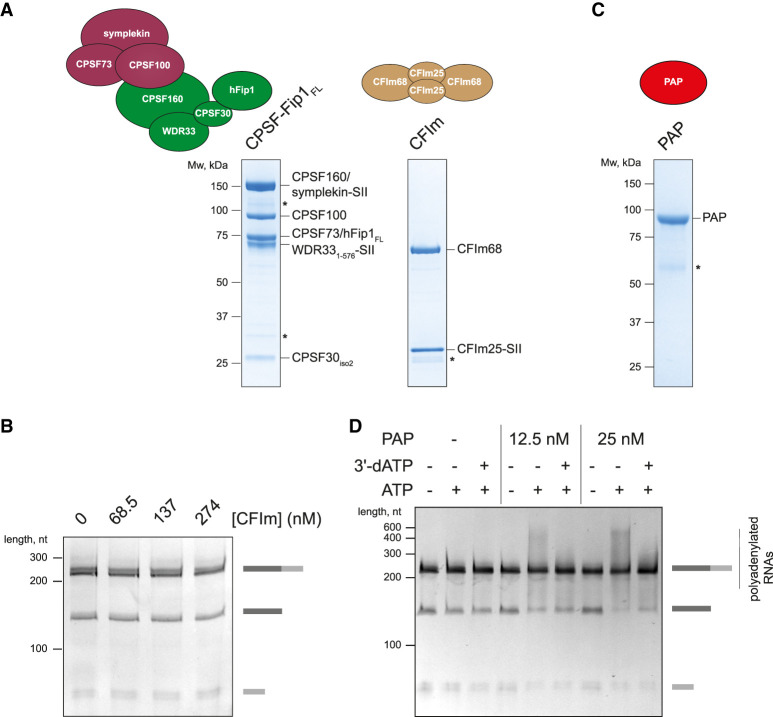
CFIm is not required for CPSF cleavage activity, and RNA is cleaved and polyadenylated in the presence of CPSF, RBBP6, CStF, CFIIm, and PAP. (*A*) SDS-PAGE analyses of purified CPSF containing full-length hFip1 (hFip1_FL_) and of purified CFIm complex. Asterisks denote degradation products. (SII) StrepII tag. (*B*) Cleavage assays of the SV40 pre-mRNA substrate with CPSF-hFip1_FL_ in the presence of increasing concentrations of CFIm. CFIm does not substantially affect CPSF cleavage activity. (*C*) SDS-PAGE analysis of purified PAP. An asterisk denotes degradation products. (*D*) Coupled cleavage and polyadenylation assays of the SV40 pre-mRNA substrate at two different concentrations of PAP in the presence of either ATP or ATP and 3′-dATP together. 3′-dATP is also called cordycepin and is known to inhibit polyadenylation. The heterogeneous products that appear in the presence of ATP are largely absent when 3′-dATP is also added. This demonstrates that polyadenylation is responsible for the diffuse band. Some substrate RNAs may also get polyadenylated by free PAP.

Since the SV40 pre-mRNA contains an upstream UGUA motif, we tested whether CFIm affected cleavage of the SV40 substrate with purified CPSF. CFIm is known to bind the RE/D domain of hFip1, which is lacking in isoform 4 of hFip1 in our CPSF complex (Zhu et al. 2018). Therefore, we also purified CPSF containing the full-length hFip1 subunit (Fig. 2A). Addition of CFIm into the cleavage assay did not provide any further stimulation of CPSF endonuclease activity in our reconstituted system (Fig. 2B; Supplemental Fig. S1A). Nevertheless, CFIm may affect cleavage in other conditions (e.g., when the concentrations of CPSF and RNA are lower) or on substrates with multiple UGUA motifs and/or multiple potential PAS sequences.

PAP is dispensable for CPSF cleavage activity, highlighting the fact that cleavage and polyadenylation can be uncoupled in vitro (Moore and Sharp 1985; Ryner and Manley 1987). Addition of PAP and ATP into a cleavage assay resulted in the polyadenylation of the 5′ cleavage product with heterogeneous poly(A) tail lengths, but PAP did not substantially change the cleavage efficiency (Fig. 2C,D; Supplemental Fig. S1B). Thus, our reconstituted CPSF complex is active in both cleavage and polyadenylation.

We also tested whether recombinant CPSF could cleave a different pre-mRNA substrate. Under the same reaction conditions, the adenoviral L3 pre-mRNA was cut with efficiency similar to that of the SV40 pre-mRNA (Supplemental Fig. S2A), suggesting that the same complement of accessory proteins (CStF, CFIIm, and RBBP6) is required for activation of the CPSF endonuclease on multiple different pre-mRNA substrates.

### Pre-mRNA cleavage by purified, recombinant CPSF is dependent on CPSF73 and a PAS

**Figure 3.**
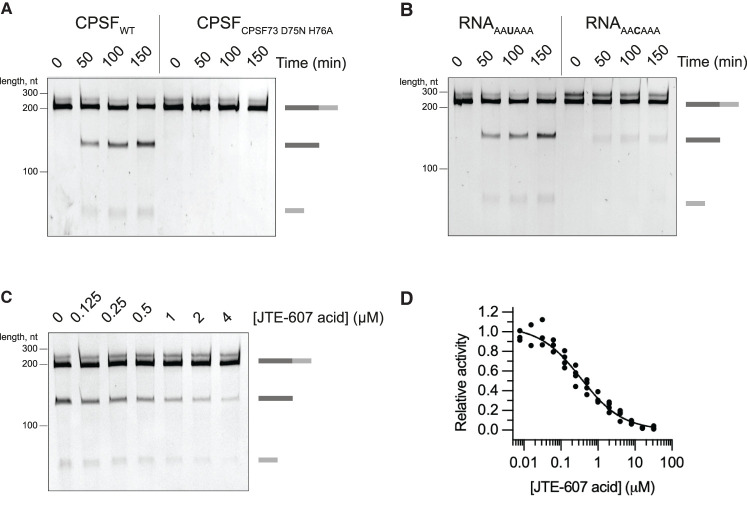
Cleavage activity of purified recombinant CPSF is dependent on CPSF73 and a PAS. (*A*) Time-course cleavage assays of the SV40 pre-mRNA substrate comparing the activities of wild-type (CPSF_WT_) and nuclease-dead (CPSF_CPSF73 D75N H76A_) CPSF complexes. (*B*) Time-course cleavage assays of SV40 pre-mRNA substrates containing either a canonical PAS (RNA_AAUAAA_) or a mutant PAS (RNA_AACAAA_) sequence. (*C*) Cleavage assays in the presence of increasing concentrations of the JTE-607 acid compound. (*D*) Dose response curve of the CPSF cleavage activity as a function of the concentration of JTE-607 acid. Each dot represents a single measurement. At least three measurements were performed for each concentration of the drug, but some points overlap.

Next, we aimed to understand the specificity of the reconstituted 3′ cleavage reaction. First, we generated a CPSF complex containing an active site mutant of CPSF73 (D75N H76A) in which the coordination of catalytic zinc ions was disrupted (Sun et al. 2020). The complex with a mutant endonuclease subunit was inactive in a cleavage assay, suggesting that the observed endonuclease activity is attributable to CPSF73 (Fig. 3A; Supplemental Fig. S2B).

We tested the activity of the mCF subcomplex alone and found that it is inactive in the absence of mPSF (Supplemental Fig. S3). The CPSF30 and WDR33 subunits within mPSF recognize the PAS sequence and contribute to specific recruitment of CPSF73 to pre-mRNAs (Clerici et al. 2018; Sun et al. 2018). Replacement of the canonical AAUAAA polyadenylation signal in the SV40 pre-mRNA with an AACAAA hexamer resulted in a substantial reduction in cleavage by CPSF (reduced by ∼80%), demonstrating that CPSF has specificity for PAS-containing RNAs (Fig. 3B). It is likely that mPSF is not only involved in RNA binding but is also required for conformational rearrangements that allow endonuclease activation (Rodríguez-Molina et al. 2021).

Recently, CPSF73 was identified as the direct target of JTE-607, a prodrug with anti-inflammatory and anticancer properties (Kakegawa et al. 2019; Ross et al. 2020). The active acid form of JTE-607 inhibits both the purified, recombinant yeast 3′ endonuclease (Ross et al. 2020) and CPSF73 within the human histone pre-mRNA 3′ end processing complex in vitro (Gutierrez et al. 2021). We therefore tested whether the JTE-607 acid analog was also inhibitory to the reconstituted human canonical 3′ end processing complex. Titrating the compound into the cleavage reaction showed dose-dependent inhibition of the endonuclease activity with an IC_50_ of ∼350 nM (Fig. 3C,D), which is very similar to the *K*_d_ of the acid form of JTE-607 for isolated CPSF73 (∼370 nM) (Ross et al. 2020). Together, these data confirm that the observed in vitro endonuclease activity is specific to CPSF73.

### Canonical and histone pre-mRNA 3′ end processing complexes are activated by different mechanisms

**Figure 4.**
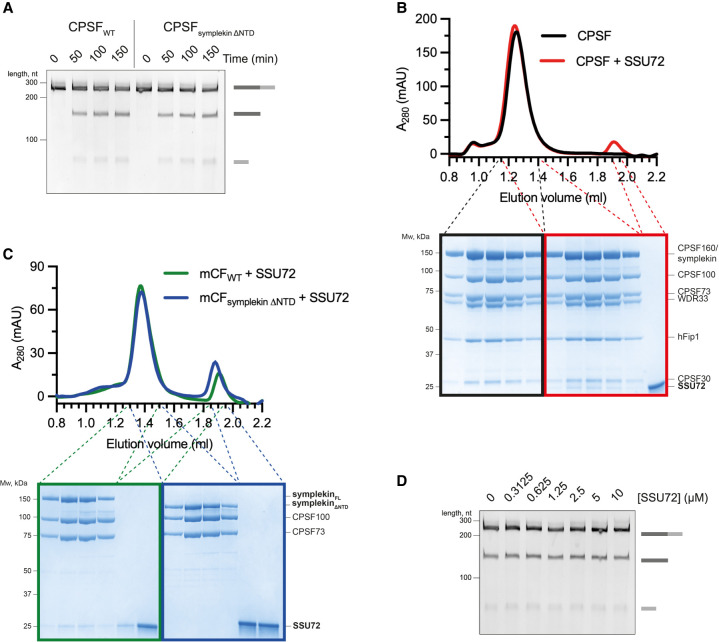
Canonical and histone pre-mRNA 3′ end processing complexes are activated by different mechanisms. (*A*) Time-course cleavage assays of the SV40 pre-mRNA substrate comparing wild-type CPSF (CPSF_WT_) and CPSF lacking the symplekin NTD (CPSF_symplekin ΔNTD_). (*B*) Gel filtration chromatograms (*top*) and SDS-PAGE analyses (*bottom*) of CPSF in the presence (red) or absence (black) of SSU72. (*C*) Gel filtration chromatograms (*top*) and SDS-PAGE analyses (*bottom*) of wild-type mCF (mCF_WT_; green) and mCF lacking the NTD of symplekin (mCF_symplekin ΔNTD_; blue) mixed with SSU72. (*D*) Cleavage assays in the presence of increasing concentrations of SSU72.

The human histone pre-mRNA 3′ processing reaction was recently reconstituted with purified proteins, and the structure of the substrate-bound complex was determined in an active state (Sun et al. 2020; Gutierrez et al. 2021). The histone processing complex shares three subunits with CPSF: symplekin, CPSF100, and CPSF73 (termed the histone cleavage complex, equivalent to mCF in CPSF). Although some aspects of endonuclease activation are carried out by proteins exclusive to the histone complex, the N-terminal domain (NTD) of symplekin (which is also found in CPSF) was shown to be essential for activating CPSF73. We tested whether the symplekin NTD plays a similar role in CPSF. To this end, we prepared a CPSF complex in which the NTD of symplekin was deleted. The CPSF complex lacking the symplekin NTD retained activity similar to that of wild-type CPSF, suggesting that the mechanism of endonuclease activation is different between the two CPSF73-containing complexes (Fig. 4A).

In addition, the phosphatase SSU72 was shown to inhibit the histone processing complex by binding to and sequestering the symplekin NTD (Sun et al. 2020). Ssu72 is a subunit of yeast CPF (Casañal et al. 2017), and hence we tested whether human SSU72 also interacts with CPSF. We found that SSU72 interacts with mCF and CPSF but not with mCF lacking the symplekin NTD (Fig. 4B,C). However, titrating SSU72 into the CPSF cleavage reaction did not affect the in vitro endonuclease activity (Fig. 4D). Together, these results suggest that, similar to the histone 3′ processing complex, SSU72 interacts with the symplekin NTD in CPSF, but the mechanism of CPSF73 activation is fundamentally different in each complex.

### RBBP6 is not a stable subunit of human CPSF

**Figure 5.**
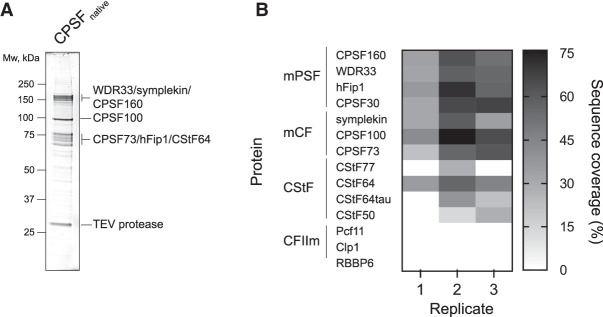
RBBP6 is not a stable subunit of CPSF purified from human cells. (*A*) SDS-PAGE analysis of the endogenous CPSF complex. The bands representing CPSF subunits are indicated. TEV protease was used to elute the complex from Strep-Tactin beads and remains present in the sample. The gel was stained with SYPRO Ruby. (*B*) Heat map representing the sequence coverage of each protein required for CPSF endonuclease activity in vitro in the endogenous CPSF preparations as detected by mass spectrometry. No RBBP6 peptides were detected across three independent experiments.

The yeast ortholog of RBBP6, Mpe1, is a constitutive subunit of the native yeast CPF complex (Vo et al. 2001; Casañal et al. 2017), but recombinant RBBP6 did not copurify with CPSF. To determine whether RBBP6 is stably associated with endogenous CPSF from human cells, we used CRISPR–Cas9 to generate a stable HEK293T cell line in which endogenous WDR33 carries a C-terminal HTBH tag (His_6_-TEV protease cleavage site–biotin acceptor peptide-His_6_) (Wang et al. 2007). The biotin acceptor peptide becomes biotinylated by endogenous enzymes in the cell, which allows the purification of CPSF on Strep-Tactin beads. We purified endogenous CPSF from the WDR33-HTBH cell line and analyzed its protein content by SDS-PAGE and mass spectrometry. The complex was relatively pure, and the enriched bands of CPSF subunits could be detected by SDS-PAGE (Fig. 5A). Mass spectrometry analysis revealed that all seven CPSF subunits copurified from human cells across multiple replicates (Fig. 5B; Supplemental Material). Among the accessory factors required for CPSF cleavage activity, only CStF subunits were pulled down by the endogenous CPSF complex. In particular, the CStF64 subunit copurified with CPSF consistently. CStF64 is associated with the native histone pre-mRNA 3′ end processing complex (Skrajna et al. 2018), and our data suggest that CStF64 may also be part of endogenous CPSF. Importantly, RBBP6 did not copurify with CPSF, consistent with a previous study (Chan et al. 2014). Therefore, despite its critical role in activating the CPSF endonuclease, RBBP6 is not a stable component of the CPSF complex.

### RBBP6 is a conserved activator of canonical pre-mRNA 3′ end cleavage

**Figure 6.**
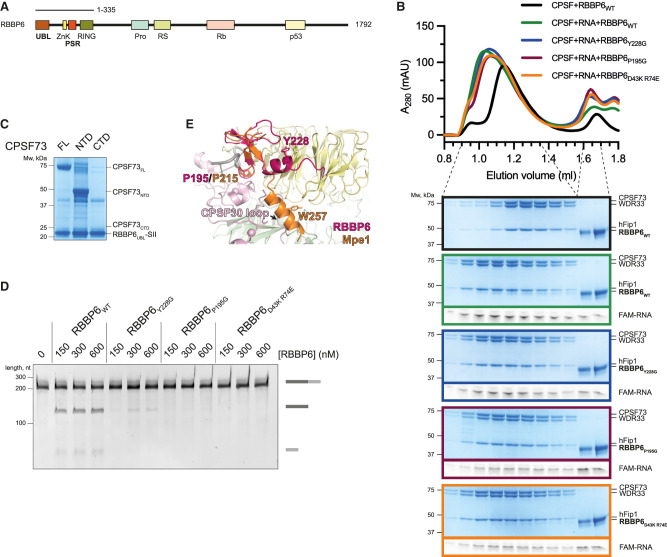
RBBP6 is a conserved activator of canonical pre-mRNA 3′ end cleavage. (*A*) Domain diagram of full-length human RBBP6 (1792 residues). The construct used in this study (residues 1–335) is indicated. (UBL) Ubiquitin-like domain, (ZnK) zinc knuckle, (PSR) pre-mRNA-sensing region, (Pro) proline-rich domain, (RS) arginine, serine-rich domain, (Rb) retinoblastoma protein-interacting region, (p53) p53-interacting region. (*B*) Gel filtration chromatograms of CPSF and RBBP6 in the presence or absence of a 5′-FAM fluorescently labeled 41-nt L3 RNA (*top*), and denaturing PAGE analysis of proteins and RNA from the indicated fractions (*bottom*). The gels are cropped and outlined in color to correspond with the colors of the chromatogram traces. (*C*) Pull-down of the SII-tagged UBL domain of RBBP6 in the presence of various constructs of CPSF73 from Sf9 insect cells. RBBP6 pulls down full-length CPSF73 and CPSF73-NTD. (FL) Full length, (NTD) N-terminal domain (residues 1–460), (CTD) C-terminal domain (residues 461–684). (*D*) Cleavage assays in the presence of various concentrations of either wild-type (RBBP6_WT_) or mutant (RBBP6_Y228G_, RBBP6_P195G_, and RBBP6_D43K R74E_) RBBP6. (*E*) Overlay of the experimental structure of the yeast Mpe1 PSR (orange) (Rodríguez-Molina et al. 2021) and an AlphaFold2 prediction of the structure of the equivalent region in human RBBP6 (magenta) overlaid on human mPSF (PDB 6BLL) (Sun et al. 2018). Residues of functional significance are indicated. A loop of CPSF30 would clash with the C-terminal helix of the Mpe1 PSR. (Yellow) WDR33, (pink) CPSF30, (green) CPSF160, (gray) PAS RNA.

Since RBBP6 is not a constitutive subunit of CPSF, we were particularly intrigued by the role of RBBP6 in activating the 3′ endonuclease. Human RBBP6 is an ∼200-kDa protein with a conserved N-terminal region containing several ordered domains, and a long, disordered, nonconserved C-terminal tail, which interacts with various binding partners that are not directly related to mRNA 3′ end processing (Fig. 6A; Supplemental Fig. S4; Sakai et al. 1995; Simons et al. 1997; Li et al. 2007; Batra et al. 2018). A construct encompassing only the N-terminal domains of RBBP6 was sufficient to stimulate CPSF (Fig. 1C), suggesting that the C-terminal region is dispensable for pre-mRNA cleavage in vitro.

To further investigate the interaction of RBBP6 and CPSF, we used size exclusion chromatography. When mixed together, RBBP6 and CPSF eluted from the column in two separate peaks, indicating that the affinity of any potential interaction is not sufficiently high for them to coelute (Fig. 6B). However, when a 41-nt fragment of L3 pre-mRNA containing a canonical PAS (AAUAAA) was included, a substoichiometric amount of RBBP6 comigrated with RNA-bound CPSF (Fig. 6B). We also performed pull-downs using MS2-tagged L3 pre-mRNA and found that RBBP6 was pulled down by RNA only in the presence of CPSF (Supplemental Fig. S5A). This suggests that RBBP6 is recruited to CPSF in an RNA-dependent manner, which is reminiscent of RNA-mediated stabilization of Mpe1 on the yeast polymerase module (Rodríguez-Molina et al. 2021). CStF and CFIIm must also be recruited to then activate cleavage.

Yeast Mpe1 contacts two subunits of CPF (Hill et al. 2019; Rodríguez-Molina et al. 2021). First, the ubiquitin-like domain (UBL) of Mpe1 stably interacts with the N-terminal nuclease domain (NTD) of the endonuclease subunit (Hill et al. 2019). The interacting residues are highly conserved, and a structure of the complex could be confidently modeled (Hill et al. 2019). An isoform of RBBP6 that contains only the UBL domain inhibits cleavage in nuclear extract by competing with full-length RBBP6 (Di Giammartino et al. 2014).

To test whether human RBBP6 and CPSF73 interact in a manner similar to the yeast proteins, we coexpressed StrepII-tagged RBBP6-UBL with various constructs of CPSF73 in insect cells and performed pull-down studies. This showed that tagged RBBP6-UBL pulled down stoichiometric amounts of both full-length CPSF73 and the CPSF73 nuclease domain (NTD) (Fig. 6C). Thus, the interaction of RBBP6-UBL and the CPSF73 nuclease is conserved in humans. However, the complex between RBBP6-UBL and the CPSF73 nuclease domain dissociated during further purification, demonstrating that the affinity between the human proteins is relatively low. We introduced mutations in RBBP6-UBL (D43K and R74E) at the putative RBBP6–CPSF73 interaction interface (Hill et al. 2019). The RBBP6-D43K-R74E mutant failed to activate CPSF in a cleavage assay, likely due to a weakened association with the CPSF–RNA complex (Fig. 6B,D). These results highlight that, like in yeast, the RBBP6-UBL contacts the CPSF73 nuclease domain. Interestingly, the interaction between RBBP6-UBL and CPSF73-NTD is stoichiometric, whereas the RBBP6_1–335_ interaction with the CPSF–RNA complex is substoichiometric. It is therefore possible that the RBBP6-UBL–CPSF73-NTD interaction is partially blocked in the context of the full CPSF complex.

We recently showed that yeast Mpe1 also binds to the Pfs2 subunit of the yeast polymerase module and directly contacts the pre-mRNA substrate (Rodríguez-Molina et al. 2021). We therefore named this region of Mpe1 the pre-mRNA-sensing region (PSR). The PSR sequence is conserved in RBBP6 and, using AlphaFold2 (Jumper et al. 2021), we predict that this region is likely to adopt an overall structure similar to that of the Mpe1 PSR (Fig. 6E; Supplemental Fig. S4). In the predicted structure, the C-terminal helix of the RBBP6 PSR is in an alternative binding position on WDR33. Interestingly, the site of Mpe1 interaction on Pfs2 is occupied by a loop of CPSF30 in the human complex, suggesting that the C-terminal helix of the RBBP6 PSR may bind to a different site on human mPSF.

To test the functional relevance of the RBBP6 PSR, we mutated a conserved aromatic residue in RBBP6, Y228. This residue is equivalent to W257 in Mpe1, which forms critical contacts with the yeast polymerase module. We also mutated P195, which contacts RNA in the yeast complex (Rodríguez-Molina et al. 2021). Both RBBP6-Y228G and RBBP6-P195G mutants were almost completely ineffective at activating the CPSF endonuclease (Fig. 6D). In addition, neither RBBP6 mutant comigrated with CPSF during gel filtration chromatography, even in the presence of RNA (Fig. 6B). Since RBBP6 and Mpe1 have been implicated in RNA binding on their own (Baejen et al. 2014; Lee and Moore 2014), we compared the relative affinities of the RBBP6 mutants for RNA using electrophoretic mobility shift assays (EMSAs). None of the mutations affected the ability of RBBP6 to bind the RNA used in gel filtration assays (Supplemental Fig. S5B), which suggests that the mutated residues are involved in RNA-dependent binding to CPSF, not in binding RNA directly. These observations demonstrate that the PSR of RBBP6 plays a crucial role in stimulating the endonuclease.

Together, these data suggest that RBBP6 interacts with CPSF in an RNA-dependent manner to act as an essential activator of the canonical pre-mRNA 3′ endonuclease. RBBP6 interactions with CPSF, and therefore the mechanism of endonuclease activation by RBBP6, are likely to be conserved from yeast to humans.

## Discussion

**Figure 7.**
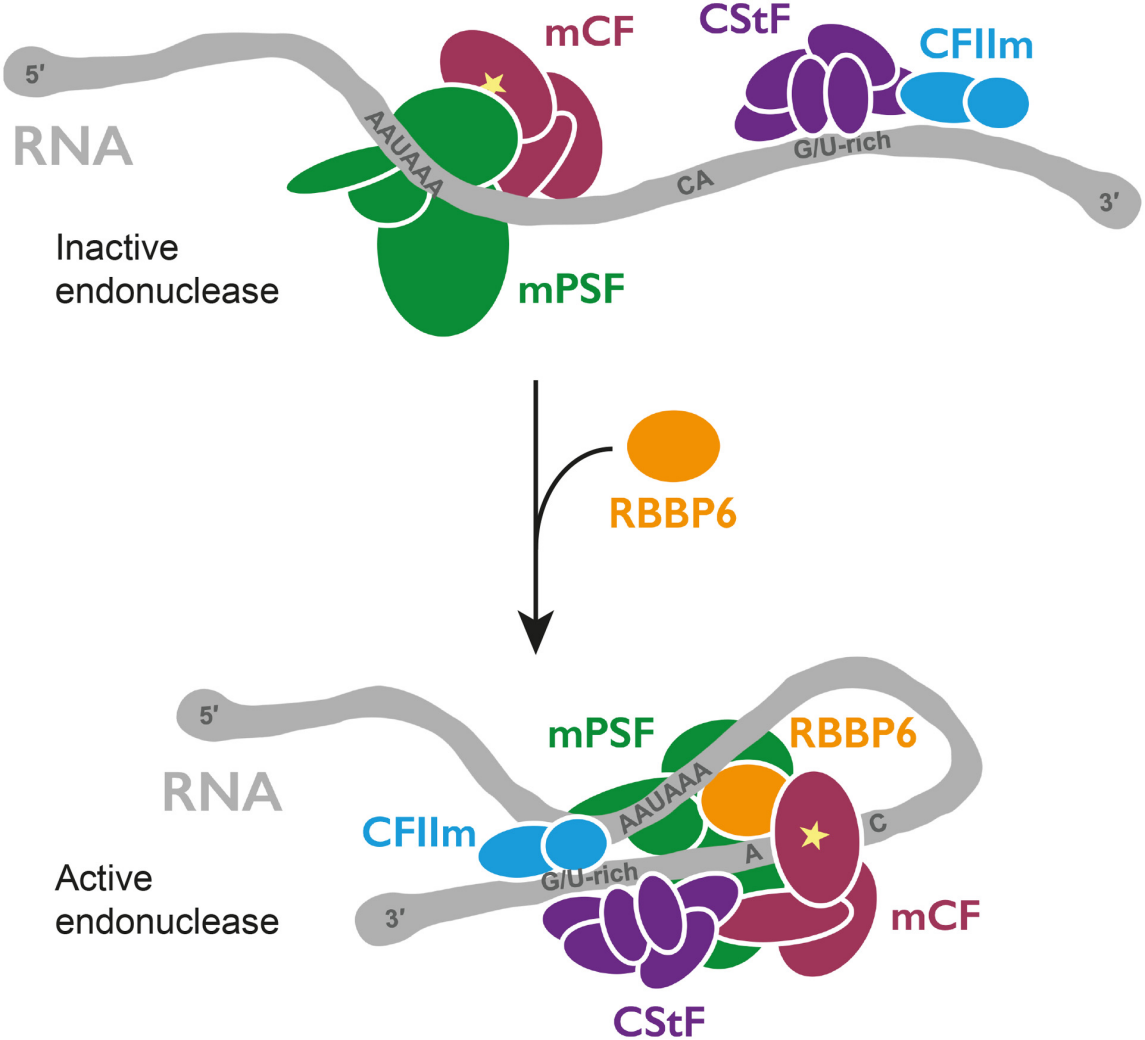
Model for activation of CPSF cleavage. Coassembly of CPSF, CStF, CFIIm, and RBBP6 activates the endonuclease CPSF73 (star). Remodeling of protein and RNA may occur.

3′ cleavage of nascent protein-coding transcripts is essential for both mRNA maturation and transcription termination. Here, we reconstituted the canonical pre-mRNA 3′ endonuclease activity of human CPSF with purified proteins and determined that CStF, CFIIm, and RBBP6 are all required for its activation (Fig. 7). Together, these four factors likely represent the minimal and universal machinery that cleaves pre-mRNAs at their 3′ ends. In agreement with this, orthologous factors (core CPF and CF IA) are required in yeast (Hill et al. 2019). However, yeast CF IB is also needed to enforce the specificity of cleavage. There is no clear ortholog of CF IB in humans.

Purified CPSF73 in isolation only weakly and nonspecifically cleaves RNA (Mandel et al. 2006). Thus, its incorporation into a seven-subunit protein complex may ensure that the endonuclease is inhibited until it is specifically activated on PAS-containing transcripts. The additional requirement for three RNA-binding accessory factors would further restrict activation, precisely positioning the endonuclease on RNA and preventing premature cleavage. In vivo, variations in nuclear concentrations of basal cleavage factors (as has been shown for CStF) (Takagaki and Manley 1998) as well as other accessory proteins (for example, CFIm) (Tseng et al. 2021) additionally regulate cleavage site selection in a transcript- and context-specific manner (Gruber and Zavolan 2019). It has been proposed that human CPSF100 may also be able to catalyze endonucleolytic cleavage (Kolev et al. 2008). However, under the conditions used here, CPSF73 is the only active endonuclease within CPSF.

Previously, RBBP6 was suggested to regulate alternative polyadenylation site selection (Di Giammartino et al. 2014), but its role has been largely underestimated, primarily because RBBP6 is not a constitutive subunit of human CPSF. In contrast, yeast Mpe1 is a core subunit of CPF (Vo et al. 2001; Casañal et al. 2017; Hill et al. 2019). Despite differences in affinity, the molecular nature of the interaction of RBBP6/Mpe1 with CPSF/CPF is likely largely conserved, as demonstrated in our mutational analyses. The affinities of other components of the 3′ end processing machineries also differ between humans and yeast. For example, the poly(A) polymerase enzyme is a constitutive subunit of the yeast but not the human complex (Kaufmann et al. 2004; Chan et al. 2014). Human CPSF has a nanomolar affinity for PAS-containing RNA (Hamilton et al. 2019), while the interaction of CPF with RNA is orders of magnitude weaker (Hill et al. 2019). In addition, human CStF and CFIIm are separate complexes, whereas in yeast they form a constitutive complex called CF IA (Gordon et al. 2011; Schäfer et al. 2018). These differences may enable alternative types of regulation of pre-mRNA 3′ end processing in different organisms while retaining the same fundamental mechanism of endonucleolytic cleavage.

RBBP6 interacts with CPSF in an RNA-dependent manner. This RNA dependence explains why RBBP6 was detected in an RNA-bound postcleavage 3′ end processing complex but not in endogenous apo CPSF (Shi et al. 2009; Chan et al. 2014). The C-terminal domain of RBBP6 is absent from our construct, and it is not required for cleavage in vitro. Interestingly, this domain contains linear peptide motifs that bind transcription factors (Rb and p53) (Saijo et al. 1995; Sakai et al. 1995; Simons et al. 1997) and also has an RS domain, which in other proteins is known to bind the spliceosome and SR proteins that regulate alternative splicing (Graveley and Maniatis 1998). Therefore, RBBP6 may help coordinate 3′ end processing with transcription and splicing in vivo.

The in vitro endonuclease activity of human CPSF is substantially slower than that of yeast CPF under similar conditions (Hill et al. 2019; Rodríguez-Molina et al. 2021). This could be due to the RNA-dependent nature of RBBP6 binding to CPSF or because additional, unknown protein factors are involved in vivo. However, it is also possible that human CPSF is an inherently inefficient and potentially more accurate endonuclease that allows more extensive regulation, for example, to enable correct cleavage site selection even on very long 3′ UTRs with multiple potential PAS sequences (Martin et al. 2012). On the other hand, CPF cleavage must be very efficient to prevent transcriptional readthrough into downstream open reading frames in yeast, where genes are closely spaced (David et al. 2006; Rodríguez-Molina et al. 2021).

The structure of the active histone pre-mRNA 3′ end processing machinery demonstrated how the propagation of conformational rearrangements across many protein factors can lead to the opening of the CPSF73 active site (Sun et al. 2020). Although we show that the precise nature of endonuclease activation differs between CPSF and the histone complex, we envision that a coordinated assembly of CPSF, CStF, CFIIm, and RBBP6 on a pre-mRNA substrate (Fig. 7) leads to a similar conformational change in CPSF73.

In this issue, Schmidt et al. (2022) also report in vitro reconstitution of the human pre-mRNA 3′ end cleavage reaction that is dependent on RBBP6. Efficient cleavage in the reconstituted system of Schmidt et al. (2022) requires the addition of ATP and PAP. While further investigation will be required to understand this, subtle difference in the assay conditions (including buffer composition, protein and RNA concentrations, and the exact protein sequences used) may account for the differences.

Inhibitors of 3′ endonucleases have been demonstrated to have anticancer (Kakegawa et al. 2019; Ross et al. 2020) and antiprotozoan (Jacobs et al. 2011; Palencia et al. 2017; Sonoiki et al. 2017; Swale et al. 2019) properties. Thus, understanding how CPSF73 is activated may aid in the development of new therapeutics for a variety of diseases. The reconstitution of human canonical pre-mRNA 3′ end processing with purified proteins provides new opportunities for studying the molecular mechanisms of cleavage and polyadenylation in detail.

## Materials and methods

### Cloning

#### CPSF, CStF, CFIIm, CFIm, RBBP6, PAP, and SSU72

*E. coli* codon-optimized genes encoding each full-length protein and isoform 2 of CPSF30 (UniProt O95639-2) in pACEBac vectors were synthesized by Epoch Life Science. All cloning was validated by sequencing (Source Bioscience). All primers and plasmids used and generated in this study are listed in Supplemental Tables S1 and S2.

To generate isoform 4 of hFip1 (UniProt Q6UN15-4), fragments containing residues 1–28 and 44–393 were amplified by PCR. Substitution F393K was also introduced during the PCR of fragment 44–393. Both fragments were assembled into an empty pACEBac vector using Gibson assembly.

To express SSU72 in *E. coli*, the coding region of SSU72 was amplified by PCR from its pACEBac vector. The forward primer contained an NdeI cleavage site, and the reverse primer had a BamHI cleavage site. After digestion with NdeI (NEB R0111) and BamHI–HF (NEB R3136) enzymes, the SSU72 coding region was ligated into an empty pET-28a vector that had been cleaved with the same enzymes. The vector contained an in-frame His_6_ tag followed by a 3C protease cleavage site on its 5′ end.

The full coding regions of CStF77, symplekin, CFIm25, and PAP were amplified by PCR from their original pACEBac vectors and cloned using Gibson assembly into pACEBac vectors containing an in-frame TEV cleavage site followed by an SII tag on their 3′ ends. For the following genes, only the sequences encoding the indicated residues were amplified by PCR: 1–576 of WDR33, 769–1555 of Pcf11, 1–335 of RBBP6, 1–142 of RBBP6 (RBBP6_UBL_), and 341–1274 of symplekin (symplekin_ΔNTD_). These fragments were also cloned into pACEBac-TEV-SII vectors as described above.

To produce catalytically inactive CPSF73 D75N H76A, the CPSF73 pACEBac plasmid was divided into three overlapping fragments, and these fragments were amplified by PCR. The mutations were located in the overlapping region between two of the three fragments. All three fragments were ligated together using Gibson assembly. To produce CPSF73_NTD_ and CPSF73_CTD_ constructs, CPSF73 residues 1–460 and 461–684, respectively, were amplified by PCR and inserted into empty pACEBac vectors using Gibson assembly.

#### Assembly into pBig1 vectors

A modified biGBac protocol was used to generate pBig1 vectors encoding all subunits of each complex (mPSF, mCF, CStF, CFIIm, CFIm, and their variants) as described previously (Weissmann et al. 2016; Hill et al. 2019).

#### CRISPR–Cas9 gene targeting in mammalian cells

Plasmids to target the 3′ end of the endogenous *WDR33* gene were a kind gift from Steven West (University of Exeter). The sequence of the HTBH tag (Wang et al. 2007) was purchased as a gBlock from IDT and inserted into a homology-directed repair plasmid by Gibson assembly.

### Protein expression

#### Baculovirus

pBig1 (mPSF, mCF, CStF, CFIIm, and CFIm) or pACEBac (RBBP6 and PAP) vectors were transformed into EMBacY cells. Extracted bacmids were transfected into Sf9 insect cells to generate the P1 virus. To produce the P2 virus, Sf9 cells were infected with the P1 virus. Proteins were overexpressed by infecting large-scale cultures of Sf9 cells (except for mPSF, which was overexpressed in Hi5 insect cells) with the P2 virus. The cells were harvested by centrifugation when the cell viability fell below ∼90% (after 3–4 d). The cell pellets were flash-frozen in liquid N_2_ and stored at −80°C. All of these procedures were described in detail previously (Hill et al. 2019; Kumar et al. 2021).

#### E. coli

*E. coli* BL21(DE3) Star cells transformed with the His_6_-SSU72-encoding pET-28a vector were grown at 37°C and then induced with 0.5 mM IPTG at OD_600_ ∼0.6 and grown overnight at 20°C. The cells were harvested by centrifugation, flash-frozen in liquid N_2_, and stored at −80°C.

### Protein purification

#### mPSF-hFip1_iso4_

A frozen cell pellet of Hi5 cells was thawed in lysis buffer [50 mM HEPES-NaOH at pH 8.0, 300 mM NaCl, 1 mM TCEP, 2 mM Mg(OAc)_2_] supplemented with 50 µg/mL DNase I, three protease inhibitor tablets (Roche 11836153001), and 1 mL of BioLock (IBA 2-0205-050) per 1 L of cell culture. The cells were lysed by sonication, and the lysate was cleared by centrifugation. The cleared lysate was filtered through a 0.65-µm filter and incubated with Strep-Tactin beads (IBA 2-1201-025) for 2–3 h. The beads were washed with lysis buffer, and the complex was eluted with 2.5 mg/mL desthiobiotin (IBA 2-1000-005) in lysis buffer. The eluate was diluted to reduce the NaCl concentration to 75 mM, filtered through a 0.45-µm filter, and applied to a 1-mL Resource Q column (Cytiva 17117701) equilibrated in buffer A [20 mM HEPES-NaOH at pH 8.0, 75 mM NaCl, 0.5 mM TCEP, 2 mM Mg(OAc)_2_]. The complex was eluted using a linear gradient of buffer B [20 mM HEPES-NaOH at pH 8.0, 1 M NaCl, 0.5 mM TCEP, 2 mM Mg(OAc)_2_] over 50 column volumes. The peak fractions were pooled, concentrated, and injected onto a Superose 6 XK 17/600-pg column (Cytiva 71501695) equilibrated in size exclusion buffer [20 mM HEPES-NaOH at pH 8.0, 150 M NaCl, 0.5 mM TCEP, 2 mM Mg(OAc)_2_]. Selected fractions were pooled and concentrated. The concentrated protein was aliquoted, flash-frozen in liquid N_2_, and stored at −80°C.

#### mPSF-hFip1_FL_

mPSF-hFip1_FL_ was purified from Hi5 cells by Strep-Tactin affinity chromatography and anion exchange chromatography as described for mPSF-hFip1_iso4_. The peak fractions of mPSF-hFip1_FL_ from a 1-mL Resource Q column were pooled, aliquoted, flash-frozen in liquid N_2_, and stored at −80°C.

#### mCF, mCF_CPSF73 D75N H76A_, and mCF_symplekin ΔNTD_

mCF and its variants were purified from Sf9 cells using the same protocol as mPSF except that (1) 50 µg/mL RNase A was added to lysis buffer, (2) buffers were supplemented with 5% (v/v) glycerol before each concentration step, and (3) size exclusion buffer contained 20 mM HEPES-NaOH (pH 8.0), 150 M NaCl, and 1 mM TCEP.

#### CStF

CStF was purified from Sf9 cells using the same protocol as mCF, except that the size exclusion buffer contained 20 mM HEPES-NaOH (pH 8.0), 200 mM NaCl, and 1 mM TCEP.

#### CFIIm

CFIIm was purified from Sf9 cells using the same protocol as mCF with a few modifications. In the lysis buffer, DNase I and RNase A were replaced by 50 U/mL benzonase (Merck E1014), and 100 µM PMSF (Merck 93482) was also added. The size exclusion buffer of CFIIm contained 20 mM Tris-HCl (pH 8.5), 150 mM NaCl, 0.5 mM TCEP, and 5% (v/v) glycerol.

#### RBBP6, RBBP6_Y228G_, RBBP6_P195G_, and RBBP6_D43K R74E_

RBBP6 was purified from Sf9 cells using the same protocol as mPSF but with different buffers: lysis buffer (50 mM HEPES-NaOH at pH 8.0, 400 mM NaCl, 1 mM TCEP), buffer A (20 mM HEPES-NaOH at pH 8.0, 40 mM NaCl, 0.5 mM TCEP), buffer B (20 mM HEPES-NaOH at pH 8.0, 1 M NaCl, 0.5 mM TCEP), and size exclusion buffer [20 mM HEPES-NaOH at pH 8.0, 200 mM NaCl, 0.5 mM TCEP, 2 mM Mg(OAc)_2_]. Also, a HiLoad 16/600 Superdex 200-pg column (Cytiva 28989335) was used for the size exclusion step.

#### CFIm

CFIm was purified from Sf9 cells by Strep-Tactin affinity chromatography and anion exchange chromatography as described for mPSF-hFip1_iso4_ but using different buffers: lysis buffer [50 mM bicine-NaOH at pH 9.0, 400 mM NaCl, 0.5 mM TCEP, 2 mM Mg(OAc)_2_, 10% (v/v) glycerol], buffer A [20 mM bicine-NaOH at pH 9.0, 150 mM NaCl, 0.5 mM TCEP, 2 mM Mg(OAc)_2_, 10% (v/v) glycerol], and buffer B [20 mM bicine-NaOH at pH 9.0, 1 M NaCl, 0.5 mM TCEP, 2 mM Mg(OAc)_2_, 10% (v/v) glycerol]. The peak fractions of CFIm from a 1-mL Resource Q column were pooled, aliquoted, flash-frozen in liquid N_2_, and stored at −80°C. Before running assays, ∼ 100 µL of CFIm was thawed and dialyzed overnight against 500 mL of dialysis buffer [20 mM bicine-NaOH at pH 9.0, 400 mM NaCl, 0.5 mM TCEP, 2 mM Mg(OAc)_2_, 10% (v/v) glycerol].

#### PAP

PAP was purified from Sf9 cells by Strep-Tactin affinity chromatography as described for mPSF-hFip1_iso4_. The eluate was incubated overnight at 4°C with 20 µg/mL TEV protease to remove the StrepII tag. The protein was further purified using a 1-mL HiTrap Q column (Cytiva 29051325) equilibrated in buffer A (50 mM HEPES-NaOH at pH 8.0, 100 mM NaCl, 1 mM TCEP) and eluted with a linear gradient of buffer B (50 mM HEPES-NaOH at pH 8.0, 1 M NaCl, 1 mM TCEP). The peak fractions were concentrated and loaded onto a HiLoad 26/600 Superdex 200-pg column (Cytiva 28989336) equilibrated in buffer containing 50 mM HEPES-NaOH (pH 8.0), 150 mM NaCl, and 1 mM TCEP. The peak fractions were pooled, concentrated, and aliquoted. The aliquots were flash-frozen in liquid N_2_ and stored at −80°C.

#### SSU72

*E. coli* cells were lysed by sonication in buffer A (50 mM HEPES-NaOH at pH 8.0, 500 mM NaCl, 1 mM TCEP, 20 mM imidazole) supplemented with two protease inhibitor tablets and 50 µg/mL DNase I. The lysate was cleared by centrifugation and loaded onto a HisTrap HP 5-mL column (Cytiva 17524701) equilibrated in buffer A. The protein was eluted with a linear gradient of buffer B (50 mM HEPES-NaOH at pH 8.0, 500 mM NaCl, 1 mM TCEP, 500 mM imidazole) over 20 column volumes. 3C protease (43 µg/mL) was added to the pooled peak fractions to remove the His_6_ tag, and the protein was dialyzed overnight using a 7-kDa cutoff membrane against dialysis buffer (50 mM HEPES-NaOH at pH 8.0, 500 mM NaCl, 1 mM DTT). The dialyzed sample was concentrated in the presence of 5% (v/v) glycerol and loaded onto a HiLoad Superdex 75 26/600 column (Cytiva 28989334) equilibrated in size exclusion buffer (20 mM HEPES-NaOH at pH 8.0, 200 mM NaCl, 1 mM TCEP). The peak fractions were concentrated in the presence of 5% (v/v) glycerol, aliquoted, and flash-frozen in liquid nitrogen. The protein was stored at −80°C.

### Preparation of RNA substrates

Sequences of all RNAs used in this study are listed in Supplemental Table S3. 5′-FAM fluorescently labeled 41-nt L3 RNA was synthesized by IDT. The DNA sequences encoding fragments of SV40 pre-mRNA with either wild-type (AAUAAA) or mutant (AACAAA) PAS were purchased as gBlocks from IDT. The sequence of the T7 RNA polymerase promoter was added to the 5′ end of the gBlock by PCR amplification.

The template of the L3 pre-mRNA was purchased from IDT as a gBlock. The fragment had a KpnI (NEB R0142) cleavage site on its 5′ end and a BamHI site on its 3′ end. After restriction digest with both enzymes, the L3 fragment was ligated into a linearized pUCIDT plasmid encoding the T7 RNA polymerase promoter followed by three MS2 loops upstream of the insert.

#### In vitro transcription

All unlabeled pre-mRNA substrates were transcribed using HiScribe T7 high-yield RNA synthesis kit (NEB E2040) and subsequently purified with Monarch RNA cleanup kit (NEB T2040).

### Cleavage assays with purified proteins

Each protein factor was first diluted in protein dilution buffer [20 mM HEPES-NaOH at pH 7.25 (measured at room temperature), 150 mM NaCl, 0.5 mM TCEP, 2 Mg(OAc)_2_]. Individually purified mPSF and mCF complexes at 2.5 µM each were mixed in protein dilution buffer and incubated for 30 min on ice. All protein components were then mixed on ice in 19 µL per condition and/or time point at the final concentrations of 50 nM CPSF, 100 nM CStF, 100 nM CFIIm, and 300 nM RBBP6. The final buffer composition of complete reactions was 20 mM HEPES-NaOH (pH 7.25; measured at room temperature), 50 mM NaCl, 0.5 mM TCEP, 2 Mg(OAc)_2_, and 1 U/µL RiboLock (Thermo EO0381). The tubes were transferred to 37°C, and the reaction was initiated by addition of the RNA substrate to a final concentration of 100 nM. Unless indicated otherwise, the reactions were stopped after 150 min by adding 5 µL of stop buffer (130 mM EDTA, 5% [v/v] SDS, 12 mg/mL proteinase K in protein dilution buffer) and incubating them for a further 15 min at 37°C. The samples were mixed with RNA gel loading dye (Thermo Scientific R0641) and loaded onto a prerun (30 W for 30 min) denaturing 10% (SV40) or 6% (L3) polyacrylamide gel containing 7 M urea in TBE buffer. The gels were run for 25 min at 400 V, stained with SYBR Green (Invitrogen S7564), and imaged using a ChemiDoc XRS+ (Bio-Rad).

The relative activity of CPSF under condition *x* was calculated as the relative intensity of the cleavage product bands in each lane relative to this ratio in control conditions (no PAP, no CFIm, or no JTE-607):

$$\mathrm{relative}\;\mathrm{activity}=\;\frac{5^{\prime }\mathrm{produc}t_{x}+3^{\prime }\mathrm{produc}t_{x}}{\mathrm{total}\;\mathrm{RN}A_{x}}\times \frac{\mathrm{total}\;\mathrm{RN}A_{0}}{5^{\prime }\mathrm{produc}t_{0}+3^{\prime }\mathrm{produc}t_{0}}.$$

The intensity values were measured in Fiji.

### Coupled cleavage and polyadenylation assay with purified proteins

Cleavage reactions were set up as described above. To test polyadenylation, PAP was added to the cleavage reaction at a final concentration of either 12.5 nM or 25 nM. 3′-dATP (Merck C9137) and/or ATP (Thermo Scientific R0441) were also included. The assays were run and analyzed as described above for cleavage-only assays.

### Sequencing of 5′ cleavage products

A standard cleavage reaction of the SV40 substrate was analyzed on a denaturing gel as described above. The band corresponding to the 5′ cleavage product was excised and submerged in 50 µL of crush and soak buffer [3 M Na(OAc) at pH 5.2, 0.1 M EDTA at pH 7.4, 20% (v/v) SDS]. The gel band was crushed with a sterile pipette tip and incubated overnight at 37°C. After taking off the supernatant, the same steps were repeated with 50 µL of fresh crush and soak buffer for 2 h. The two supernatants were combined, and the extracted RNA was precipitated for 2 h at −20°C in 300 µL of absolute ethanol with 1 µL of Glycoblue (Invitrogen AM9516). The RNA was pelleted in a chilled microcentrifuge at maximum speed for 10 min and washed with 500 µL of 70% ethanol. The RNA pellet was resuspended in 20 µL of DEPC water. An adenylated adaptor of a known sequence was ligated to the 3′ end of the extracted 5′ cleavage product using T4 RNA ligase 2, truncated (NEB M0242). The RNA was purified from the ligation reaction components using Monarch RNA cleanup kit. The 5′ cleavage products that contained the adaptor were converted into cDNA using SuperScript IV first strand synthesis system (Invitrogen 18091050) with a forward primer specific to a 5′ region of the SV40 RNA and a reverse primer that anneals to the adaptor. The cDNA was further amplified by PCR and ligated into a bacterial vector using Zero Blunt PCR cloning kit (Invitrogen K270040). After transformation into TOP10 *E. coli* cells, 15 colonies were picked, and the isolated plasmids were sequenced using the M13R primer (Source Bioscience) to determine the 3′ end of the 5′ cleavage product.

### Assays with JTE-607 acid compound

The prodrug of JTE-607 was purchased from Tocris and hydrolyzed to JTE-607 acid analog as previously described (Ross et al. 2020; Gutierrez et al. 2021). Standard cleavage assays were set up in the presence of various concentrations of the acid form of JTE-607, and the samples were analyzed by denaturing polyacrylamide gel electrophoresis as described above. The quantitation data were plotted in Prism 9 and fitted to the equation of “[inhibitor] versus response − variable slope (four parameters)” with an R^2^ value of 0.9656.

### Endogenous pull-downs from mammalian cells

A stable HEK293T cell line in which the endogenous WDR33 subunit carried a C-terminal HTBH tag was generated using an established protocol for CRISPR–Cas9-based gene targeting (Eaton et al. 2018). The correct clones were identified by sequencing and Western blotting.

HEK293T cells were grown on 150-mm dishes in high-glucose GlutaMAX DMEM medium (Gibco 10566016) supplemented with 10% fetal bovine serum and penicillin–streptamycin. Native CPSF was purified from either total cell extract (replicate 1) or nuclear extract (replicates 2 and 3).

In experiment 1, the HEK293T-WDR33-HTBH cells were harvested using a cell scraper, washed in PBS, and resuspended in hypotonic lysis buffer [20 mM HEPES-NaOH at pH 8.0, 2 mM Mg(OAc)_2_, 2 mM EDTA, 1 mM EGTA, 1 mM DTT, 10% glycerol] supplemented with protease inhibitor tablets and 100 µM PMSF. Total cell extract was prepared by freeze–thaw lysis before adjusting the NaCl concentration to 300 mM. The lysate was clarified by centrifugation and incubated with Strep-Tactin beads. The beads were washed in buffer containing 50 mM HEPES-NaOH (pH 8.0), 300 mM NaCl, 1 mM DTT, 2 mM Mg(OAc)_2_, and 10% (v/v) glycerol, and the complex was eluted from the beads in the same buffer by cleavage with TEV protease. TEV protease remained in the eluted sample.

In experiment 2, nuclear extract of the HEK293T-WDR33-HTBH cell line was prepared using homogenization. The cell pellet was resuspended in hypotonic lysis buffer (10 mM HEPES-KOH at pH 7.9, 10 mM KCl, 1 m DTT, 1.5 mM MgCl_2_) supplemented with protease inhibitor tablets and 100 µM PMSF. The cells were incubated on ice, and the intact nuclei were isolated by centrifugation. The pellet containing the nuclei was resuspended in extraction buffer (20 mM HEPES-KOH at pH 7.9, 420 mM KCl, 1 m DTT, 1.5 mM MgCl_2,_ 0.2 mM EDTA, 25% [v/v] glycerol). The nuclei were lysed by homogenization, and the nuclear extract was clarified by centrifugation. The breakdown of the nuclei was checked by Trypan blue staining. The extract was diluted to the final KCl concentration of 300 mM before applying the sample to Strep-Tactin beads. CPSF was purified as described in experiment 1.

In experiment 3, nuclear extract of the HEK293T-WDR33-HTBH cell line was prepared using detergent lysis. The cell pellet was resuspended in lysis buffer [10 mM HEPES-KOH at pH 8.0, 100 mM KCl, 2 mM Mg(OAc)_2_, 0.3 M sucrose, 0.2% (v/v) Igepal (Merck I3021), 1 mM TCEP]. The cells were incubated on ice, and the intact nuclei were isolated by centrifugation. The pellet containing the nuclei was resuspended in extraction buffer [20 mM HEPES-KOH at pH 8.0, 300 mM KCl, 2 mM Mg(OAc)_2_, 10% (v/v) glycerol, 0.2% (v/v) Igepal, 1 mM TCEP]. The breakdown of the nuclei was checked by Trypan blue staining. The nuclear extract was clarified by centrifugation, and the sample was applied to Strep-Tactin beads. CPSF was purified as described in experiment 1.

The eluate from each experiment was analyzed by SDS-PAGE. The gels were stained with SYPRO Ruby (Invitrogen S12000). The gel in Figure 5A came from experiment 1. The samples were also subjected to protein identification by tandem mass spectrometry. Mass spectrometry data were analyzed using Scaffold4 software.

### Pull-downs from insect cells

A P2 virus encoding RBBP6_UBL_-SII and a P2 virus carrying a gene of one of the CPSF73 variants (CPSF73_FL_, CPSF73_NTD_, or CPSF73_CTD_) were used to coinfect Sf9 cells at ∼2 million cells/mL. The cultures were harvested after 3 d by centrifugation and washed in ice-cold PBS. The cell pellets were lysed using glass beads (Merck G8772) in lysis buffer [50 mM HEPES-NaOH at pH 8.0, 300 mM NaCl, 1 mM TCEP, 2 mM Mg(OAc)_2_] supplemented with two protease inhibitor tablets per 50 mL of buffer. The lysates were cleared by centrifugation and applied to Strep-Tactin beads. After a 2-h incubation, the beads were washed in lysis buffer, and the bound proteins were eluted by incubating the samples in NuPAGE LDS sample buffer (Invitrogen NP0007) for 2 min at 98°C. The eluted proteins were analyzed on a NuPAGE 4%–12% Bis-Tris 1.0-mm mini protein gel (Invitrogen NP0321), and the gel was stained with Instant Blue (Abcam 119211).

### Gel filtration chromatography

All samples were incubated for 30 min on ice before analysis. To investigate RBBP6 binding to CPSF, 2.5 µM CPSF and 7.5 µM RBBP6 or its point mutants were mixed with or without 5 µM 5′-FAM 41-nt L3 RNA. The CPSF-RBBP6 samples were loaded onto a Superose 6 Increase 3.2/300 column (Cytiva 29091598) equilibrated in HEPES-NaOH (pH 8.0), 50 mM NaCl, and 0.5 mM TCEP. To test SSU72 binding to CPSF and mCF variants, 2.5 µM CPSF/mCF/mCF_symplekin ΔNTD_ was incubated with 10 µM SSU72. The samples were loaded onto the same column but in a buffer containing HEPES-NaOH (pH 8.0), 150 mM NaCl, and 0.5 mM TCEP. The protein content of the peak fractions was analyzed by SDS-PAGE as described above. To detect the RNA, stop buffer was added to an aliquot of each fraction. After incubation for 10 min at 37°C, RNA loading dye was added, and the samples were loaded onto 15% Novex TBE-urea gels (300 V, 50 min). The gels were scanned using a FAM channel on a Typhoon FLA 7000 instrument (GE Healthcare).

### In vitro pull-downs on M2-L3 pre-mRNA

The pull-downs were performed in pull-down buffer containing 20 mM HEPES-NaOH (pH 8.0), 50 mM NaCl, 0.5 mM TCEP, and 2 mM Mg(OAc)_2_. First, 520-nt MS-L3 pre-mRNA was incubated with MBP-tagged MS2 protein at molar ratio 1:3 for 45 min on ice. Then, 3 µM RBBP6, 1 µM CPSF, or 3 µM RBBP6 + 1 µM CPSF was added and incubated for 1.5 h. The mixture containing RBBP6/CPSF/RBBP6 + CPSF and MBP-MS2-bound L3 pre-mRNA was mixed with amylose beads (NEB E8021) equilibrated in pull-down buffer and incubated rotating for 1.5 h at 4°C. The beads were washed with pull-down buffer. Protein–RNA complexes were eluted in pull-down buffer supplemented with 20 mM maltose (Merck 63418). The eluates were loaded onto a NuPAGE 4%–12% Bis-Tris 1.0-mm mini protein gel. The proteins were transferred onto a nitrocellulose membrane using Trans-Blot Turbo transfer system (Bio-Rad 1704158). StrepII-tagged proteins (RBBP6, symplekin, and WDR33) were detected using streptavidin-HRP conjugate (Merck Millipore 18152) and Amershan ECL detection reagents (Cytiva RPN2106). The blots were visualized using a ChemiDoc XRS+ (Bio-Rad).

### Electrophoretic mobility shift assays (EMSAs)

Indicated concentrations of various point mutants of RBBP6 were mixed with 100 nM 41-nt 5′-FAM-labeled L3 pre-mRNA and orange G loading dye (0.4% [w/v] orange G, 50% [v/v] glycerol, 1 mM EDTA). The protein–RNA mixtures were incubated for 15 min on ice and then loaded onto a 10% native polyacrylamide gel. The gel was run for 50 min at 100 V at 4°C. The RNA was visualized using a FAM channel on a Typhoon FLA 7000 instrument (GE Healthcare).

## Supplementary Material

Supplemental Material
