# Supplementary material for: RBBP6 activates the pre-mRNA 3′ end processing machinery in humans
Source: Genes Dev. 2022 Feb 1;36(3-4):210–24. doi: 10.1101/gad.349223.121 (PMC8887125; doi:10.1101/gad.349223.121)
Supplement: Supplemental Material [file supp_36_3-4_210__DC1.html]

RBBP6 activates the pre-mRNA 3′ end processing machinery in humans — Supplemental Material 

# RBBP6 activates the pre-mRNA 3′ end processing machinery in humans

## Supplemental Material

- Supplemental\_Figures\_Tables.pdf
- Supplemental\_Material\_Replicates.xlsx
