# Supplementary material for: RBBP6 activates the pre-mRNA 3′ end processing machinery in humans
Source: Genes Dev. 2022 Feb 1;36(3-4):210–24. doi: 10.1101/gad.349223.121 (PMC8887125; doi:10.1101/gad.349223.121)
Supplement: Supplemental Material [file supp_gad.349223.121_Supplemental_Figures_Tables.pdf]

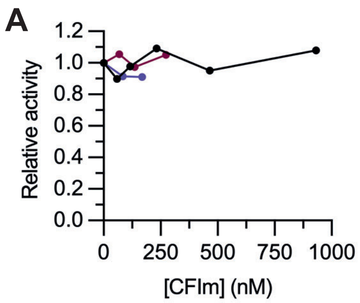

Supplemental Fig. S1. CFIm and PAP do not substantially affect CPSF cleavage activity under our assay conditions. (*A*) Quantification of relative endonuclease activity across a range of CFIm concentrations from three independent experiments (black, purple, maroon). (*B*) Quantification of relative endonuclease activity across a range of PAP concentrations from three independent experiments (black, purple, maroon). In both cases, the relative activity does not vary much across the concentrations tested, and there is no clear trend in the activity as the concentration of the indicated proteins increases.

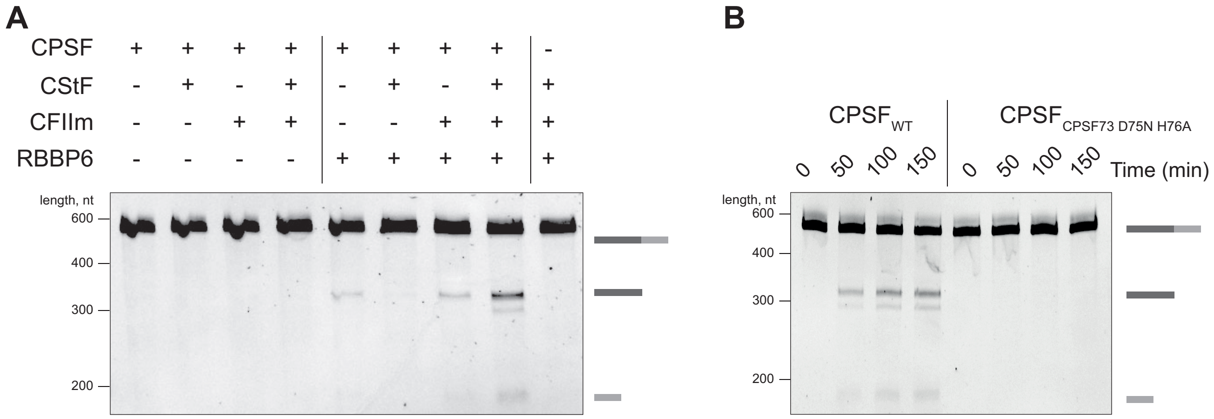

Supplemental Fig. S2. L3 pre-mRNA substrate is cleaved by purified CPSF. (*A*) Denaturing gel electrophoresis of the MS2-tagged L3 520-nt pre-mRNA substrate after incubation with various combinations of human 3′-end processing factors. For both L3 and SV40 (Figure 1C), there is a small amount of cleavage by CPSF + RBBP6 and CPSF + RBBP6 + CFIIm but the CPSF complex is substantially activated with RBBP6, CFIIm and CStF. (*B*) Time-course cleavage assays of the MS2-L3 pre-mRNA substrate comparing wild-type ($CPSF_{WT}$) and nuclease-dead ($CPSF_{CPSF73\ D75N\ H76A}$) CPSF complexes.

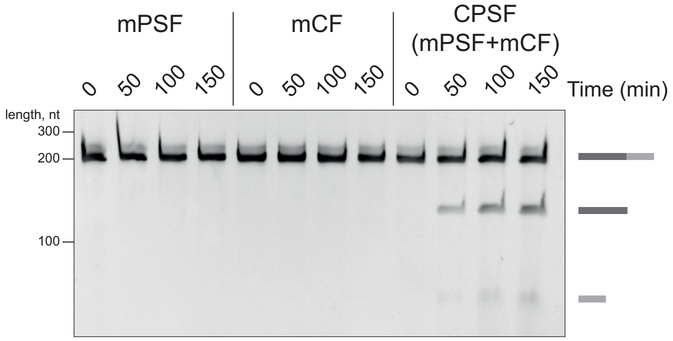

Supplemental Fig. S3. CPSF73 is inactive in the absence of mPSF. Time-course cleavage assays of the SV40 pre-mRNA substrate comparing activities of mPSF, mCF and CPSF (mPSF and mCF combined).

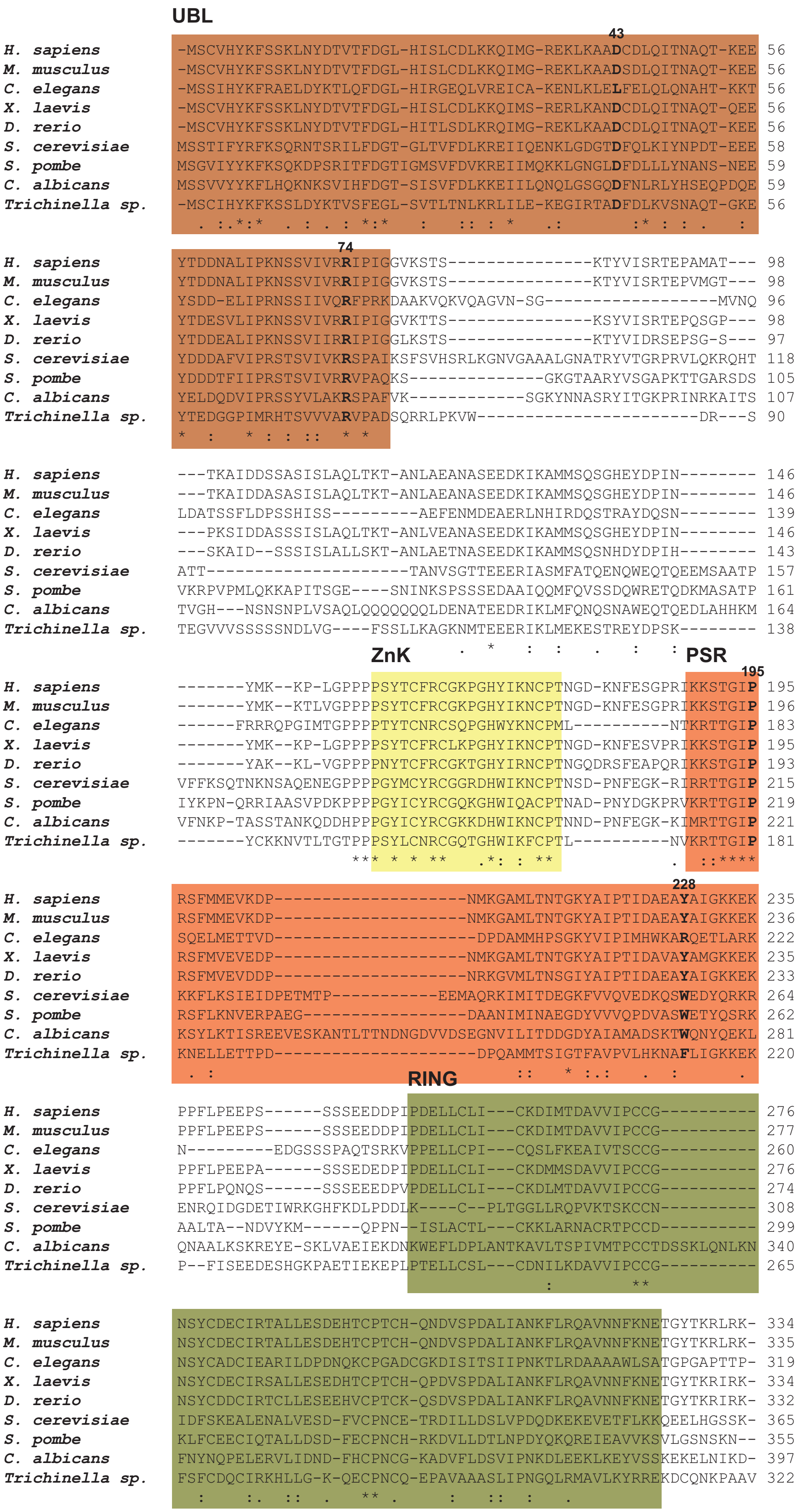

UBL
43
H. sapiens -MSCVHYKFSSKLNYDTVTFDGL-HISLCDLKKQIMG-REKLKAADCDLQITNAQT-KEE 56
M. musculus -MSCVHYKFSSKLNYDTVTFDGL-HISLCDLKKQIMG-REKLKAADSDLQITNAQT-KEE 56
C. elegans -MSSIHYKFRAELDYKTLQFDGL-HIRGEQLVREICA-KENLKLELFELQLQNAHT-KKT 56
X. laevis -MSCVHYKFSSKLNYDTVTFDGL-HISLCDLKKQIMS-RERLKANDCDLQITNAQT-QEE 56
D. rerio -MSCVHYKFSSKLNYDTVTFDGL-HITLSDLKRQIMG-REKLKAADCDLQITNAQT-KEE 56
S. cerevisiae MSSTIFYRFKSQRNTSRILFDGT-GLTVFDLKREIIQENKLGDGTDFQLKIYNPDT-EEE 58
S. pombe MSGVIYYKFKSQKDPSRITFDGTIGMSVFDVKREIIMQKKLGNGLDFDLLLYNANS-NEE 59
C. albicans MSSVVYYKFLHQKNKSVIHFDGT-SISVFDLKKEIILQNQLGSGQDFNLRLYHSEQPDQE 59
Trichinella sp. -MSCIHYKFKSSLDYKTVSFEGL-SVTLTNLKRLILE-KEGIRTADFDLKVSNAQT-GKE 56
74
H. sapiens YTDDNALIPKNSSVIVRRIPIGGVKSTS---------------KTYVISRTEPAMAT--- 98
M. musculus YTDDNALIPKNSSVIVRRIPIGGVKSTS---------------KTYVISRTEPVMGT--- 98
C. elegans YSDD-ELIPRNSSIIVQRFPRKDAAKVQKVQAGVN-SG------------------MVNQ 96
X. laevis YTDESVLIPKNSSVIVRRIPIGGVKTTS---------------KSYVISRTEPQSGP--- 98
D. rerio YTDDEALIPKNSSVIIRRIPIGGLKSTS---------------KTYVIDRSEPSG-S--- 97
S. cerevisiae YDDDAFVIPRSTSVIVKRSPAIKSFSVHSRLKGNVGAAALGNATRYVTGRPRVLQKRQHT 118
S. pombe YDDDTFIIPRSTSVIVRRVPAQKS--------------GKGTAARYVSGAPKTTGARSDS 105
C. albicans YELDQDVIPRSSYVLAKRSPAFVK------------SGKYNNASRYITGKPRINRKAITS 107
Trichinella sp. YTEDGGPIMRHTSVVVARVPADSQRRLPKVW----------------------DR---S 90
H. sapiens ---TKAIDDSSASISLAQLTKT-ANLAEANASEEDKIKAMMSQSGHEYDPIN-------- 146
M. musculus ---TKAIDDASASISLAQLTKT-ANLAEANASEEDKIKAMMSQSGHEYDPIN-------- 146
C. elegans LDATSSFLDPSSHISS---------AEFENMDEAERLNHIRDQSTRAYDQSN-------- 139
X. laevis ---PKSIDDASSSISLAQLTKT-ANLVEANASEEDKIKAMMSQSGHEYDPIN-------- 146
D. rerio ---SKAID--SSSISLALLSKT-ANLAETNASEEDKIKAMMSQSNHDYDPIH-------- 143
S. cerevisiae ATT--------------------TANVSGTTEEERIASMFATQENQWEQTQEEMSAATP 157
S. pombe VKRPVPMLQKKAPITSGE----SNINKSPSSSEDAAIQQMFQVSSDQWRETQDKMASATP 161
C. albicans TVGH---NSNSNPLVSAQLQQQQQQQLDENATEEDRIKLMFQNQSNAWEQTQEDLAHHKM 164
Trichinella sp. TEGVVVSSSSSNDLVG----FSSLLKAGKNMTEEERIKLMEKESTREYDPSK-------- 138
ZnK
PSR
195
H. sapiens -------YMK--KP-LGPPPPSYTCFRCGKPGHYIKNCPTNGD-KNFESGPRIKKSTGIP 195
M. musculus -------YMK--KTLVGPPPPSYTCFRCGKPGHYIKNCPTNGD-KNFESGPRIKKSTGIP 196
C. elegans ------FRRRQPGIMTGPPPPTYTCNRCSQPGHWYKNCPML----------NTKRTTGIP 183
X. laevis -------YMK--KP-LGPPPPSYTCFRCLKPGHYIKNCPTNGD-KNFESVPRIKKSTGIP 195
D. rerio -------YAK--KL-VGPPPPNYTCFRCGKTGHYIRNCPTNGQDRSFEAPQRIKKSTGIP 193
S. cerevisiae VFFKSQTNKNSAQENEGPPPPGYMCYRCGGRDHWIKNCPTNSD-PNFEGK-RIRRTTGIP 215
S. pombe IYKPN-QRRIAASVPDKPPPPGYICYRCGQKGHWIQACPTNAD-PNYDGKPRVKRTTGIP 219
C. albicans VFNKP-TASSTANKQDDHPPPGYICYRCGKKDHWIKNCPTNND-PNFEGK-KIMRTTGIP 221
Trichinella sp. -------YCKKNVTLTGTPPPSYLCNRCGQTGHWIKFCPTL----------NVKRTTGIP 181
228
H. sapiens RSFMMEVKDP--------------------NMKGAMLTNTGKYAIPTIDAEAYAIGKKEK 235
M. musculus RSFMMEVKDP--------------------NMKGAMLTNTGKYAIPTIDAEAYAIGKKEK 236
C. elegans SQELMETTVD---------------------DPDAMMHPSGKYVIPIMHWKARQETLARK 222
X. laevis RSFMVEVEDP--------------------NMKGAMLTNTGKYAIPTIDAVAYAMGKKEK 235
D. rerio RSFMVEVDDP--------------------NRKGVMLTNSGIYAIPTIDAEAYAIGKKEK 233
S. cerevisiae KKFLKSIEIDPETMTP------------EEMAQRKIMITDEGKFVVQVEDKQSWEDYQRKR 264
S. pombe RSFLKNVERPAEG-----------------DAANIMINAEGDYVVVQPDVASWETYQSRK 262
C. albicans KSYLKTISREEVESKANTLTTNDNGDVVDSEGNVILITDDGDYAIAMADSKTWQNYQEKL 281
Trichinella sp. KNELLETTPD---------------------DPQAMMTSIGTFAVPVLHKNAFLIGKKEK 220
RING
H. sapiens PPFLPEEPS------SSSEEDDPIPDELLCLI---CKDIMTDAVVIPCCG---------- 276
M. musculus PPFLPEEPS------SSSEEDDPIPDELLCLI---CKDIMTDAVVIPCCG---------- 277
C. elegans N---------EDGSSSPAQTSRKVPPELLCPI---CQSLFKEAIVTSCCG---------- 260
X. laevis PPFLPEEPA------SSSEDEDPIPDELLCLI---CKDMMSDAVVIPCCG---------- 276
D. rerio PPFLPQNQS------SSSEEEDPVPDELLCLI---CKDLMTDAVVIPCCG---------- 274
S. cerevisiae ENRQIDGDETIWRKGHFKDLPDDLK----C--PLTGGLLRQPVKTSKCCN---------- 308
S. pombe AALTA--NDVYKM------QPPN--ISLACTL---CKKLARNACRTPCCD---------- 299
C. albicans QNAALKSKREYE-SKLVAEIEKDNKWEFLDPLANTKAVLTSPIVMTPCCTDSSKLQNLKN 340
Trichinella sp. P--FISEEDESHGKPAETIEKEPLPTELLCSL---CDNILKDAVVIPCCG---------- 265
H. sapiens NSYCDECIRTALLESDEHTCPTCH-QNDVSPDALIANKFLRQAVNNFKNETGYTKRLRK- 334
M. musculus NSYCDECIRTALLESDEHTCPTCH-QNDVSPDALIANKFLRQAVNNFKNETGYTKRLRK- 335
C. elegans NSYCADCIEARILDPDNQKCPGADCGKDISITSIIPNKTLRDAAAAWLSATGPGAPTTP- 319
X. laevis NSYCDECIRSALLESEDHTCPTCH-QPDVSPDALIANKFLRQAVNNFKNETGYTKRIRK- 334
D. rerio NSYCDDCIRTCLLESEEHVCPTCK-QSDVSPDALIANKFLRQAVNNFKNETGYTKRIRK- 332
S. cerevisiae IDFSKEALENALVESD-FVCPNCE-TRDILLDSLVPDQDKEKEVETFLKKQEELHGSSK- 365
S. pombe KLFCEECIQTALLDSD-FECPNCH-RKDVLLDTLNPDYQKQREIEAVVKSVLGSNSKN-- 355
C. albicans FNYNQPELERVLIDND-FHCPNCG-KADVFLDSVIPNKDLEEKLKEYVSSKEKELNIKD- 397
Trichinella sp. FSFCDQCIRKHLLG-K-QECPNCQ-EPAVAAASLIPNGQLRMAVLKYRREKDCQNKPAAV 322

Supplemental Fig. S4. Sequence alignment of RBBP6 orthologs. Residues corresponding to human RBBP6 1-335 were aligned to orthologs from other eukaryotes. Residues investigated in this study are indicated. UBL - ubiquitin-like domain; ZnK - zinc knuckle; PSR - pre-mRNA-sensing region. * - conserved amino acid identity; : - amino acid properties conserved; . - properties partly conserved. The alignment was generated with Uniprot.

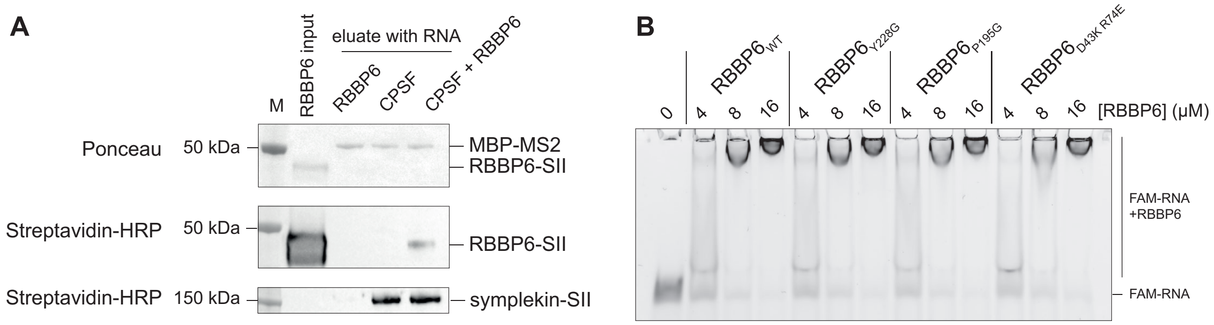

Supplemental Fig. S5. CPSF recruits RBBP6 to pre-mRNAs. (*A*) *In vitro* pull-downs of RBBP6 by MS2-tagged 520-nt L3 pre-mRNA substrate in the presence and absence of CPSF. The RNA was mixed with MBP-tagged MS2 and the indicated proteins, and immobilized on amylose beads. The eluates were analyzed by Western blots against the StrepII tag. Under these experimental conditions, RBBP6 is recruited to RNA only in the presence of CPSF. M - protein molecular weight marker. (*B*) Electrophoretic mobility shift assays (EMSAs) of the binding to a 5′-FAM fluorescently-labelled 41-nt L3 RNA by RBBP6 and its point mutants. The mutations do not affect RBBP6 binding to RNA in the absence of CPSF.

Supplemental Table S1: Oligonucleotides used in this study

Name	Sequence
**Tagging**	
CStF77_F	CCGTCCCACCATCGGGCGCGGATCCATGTCTGGTGATGGTGCAACCG
CStF77_R	CCTGGAAATACAGGTTTTCCTCGAGGCGAATACGTTTCTGCTGACGT
symplekin_F	CCGTCCCACCATCGGGCGCGGATCCATGGCATCTGGTTCTGGTGATT
symplekin_R	CCTGGAAATACAGGTTTTCCTCGAGGCTGTTGCCCTTCGCTTCCGGT
WDR33_1-572_F	CCGTCCCACCATCGGGCGCGGATCCATGGCAACTGAAATCGGTTCTC
WDR33_1-572_R	CCTGGAAATACAGGTTTTCCTCGAGGATCTGTTCTACCTGCTTCTG
Pcf11_DN769_F	CCGTCCCACCATCGGGCGCGGATCCATGTTTGAAGGTCCAAACAAGCTGAG
hPcf11_R	CCTGGAAATACAGGTTTTCCTCGAGCACAGATTCCACGGTATCGTTC
RBBP6_1-142_F	CCGTCCCACCATCGGGCGCGGATCCATGTCCTGTGTTCATTACAAG
RBBP6_1-335	CCTGGAAATACAGGTTTTCCTCGAGCTGTTTACGCAGACGTTTAG
RBBP6_1-142_R	CCTGGAAATACAGGTTTTCCTCGAGGTATTCGTGGCCAGACTGGCTC
symp_64_F	CCGTCCCACCATCGGGCGCGGATCCATGTCCAGCAAAGATACCCGTAAG
CFIm25_F	CCGTCCCACCATCGGGCGCGGATCCATGTCTGTTGTTCCACCGAATC
CFIm25_R	CCTGGAAATACAGGTTTTCCTCGAGGTTGTAGATGAAGTTGAAGCGA
PAP_F	CCGTCCCACCATCGGGCGCGGATCCATGCCATTTCCAGTTACTACTC
PAP_R	CCTGGAAATACAGGTTTTCCTCGAGGCGATTCAGACGCAGTTTGATG
SSU72_pET_F	GCGCGCCATATGCCAAGCTCTCCGCTGCGTGTAGCTG
SSU72_pET_R	GCGCGCGGATCCTTAGTAGAAGCATACGGTGTGCAGG
**hFip1iso$_4$**	
Fip1_iso4_F1	CCGTCCCACCATCGGGCGCGGATCCATGTCTGCAGGTGAGGTTGAACG
Fip1_iso4_R1	CGTTCCACTTCGTTCTCGTCACCATACAGCCATTCTTCC
Fip1_iso4_F2	GGAAGAATGGCTGTATGGTGACGAGAACGAAGTGGAACG
Fip1_iso4_R2	GCTTGTCGAGACTGCAGGCTCTAGATTATTTACCTGGTGGTGGAATCAGCGG
**CPSF73 constructs**	
73mut_F1	CACTTCCACCTGAACGCGTGCGGTGCCCTG
73mut_R1	TCTTGCCATCCACGGTCACGGACAGAATAG
73mut_F2	CTATTCTGTCCGTGACCGTGGATGGC
73mut_R2	GGCCGCGGAGTTGTTCGGTAAATTGTCAC
73mut_F3	GTGACAATTTACCGAACAACTCCGCGGCC
73mut_R3	GCAGGGCACCGCACGCGTTCAGGTGGAAGTG
CPSF73_1-460_F	CCGTCCCACCATCGGGCGCGGATCCATGTCCGCAATCCCAGCTGAAG
CPSF73_1-460_R	GCTTGTCGAGACTGCAGGCTCTAGATTAACCACGGAAGTTCAGAGTCAC
CPSF73_C_F	GACTCTAGCGTTTAAACTTAAGCTTGTTATGGGCTCCGCAATCCCAGCTGAAG
CPSF73_C_R	CTTGAAACAGAACTTCCAGGGATCCGTGCACTGGAGTCAGCGCTTC
**RNA production and sequencing**	
VB304_SV40_F	TATGCTAATACGACTCACTATAGGGAGACATGATAAGATACATTGATG
VB287_SV40_R	ATCCGTCGAGGTTTTACTTGC
SV40_seq_F	GGACAAACCACAACTAGAATGCAG
RT Adaptor	GTGGGCTCTTAAGGT
30nt_M13R	AGCGGATAACAATTTCACACAGGA

**pBig1 assembly**	
pB_pIDC_CasI_F	AACGCTCTATGGTCTAAAGATTTAAATCGACCTACTCCGGAATATTAATAGATC
pB_pIDC_CasI_R	AAACGTGCAATAGTATCCAGTTTATTTAAATGGTTATGATAGTTATTGCTCAGCG
pB_pIDC_CasII_F	AAACTGGATACTATTGCACGTTTAAATCGACCTACTCCGGAATATTAATAGATC
pB_pIDC_CasII_R	AAACATCAGGCATCATTAGGTTTATTTAAATGGTTATGATAGTTATTGCTCAGCG
pB_pIDC_CasIII_F	AAACCTAATGATGCCTGATGTTTAAATCGACCTACTCCGGAATATTAATAGATC
pB_pIDC_CasIII_R	AAACTAAGCTATGTGAACCGTTTATTTAAATGGTTATGATAGTTATTGCTCAGCG
pB_pIDC_CasIV_F	AAACGGTTCACATAGCTTAGTTTAAATCGACCTACTCCGGAATATTAATAGATC
pB_pIDC_Casw_R	AACCCCGATTGAGATATAGATTTATTTAAATGGTTATGATAGTTATTGCTCAGCG
**CRISPR-Cas9 gene targeting**	
WDR33_repair_lin_F	GGATCAGGGGCCACTAACTT
WDR33_repair_lin_R	CCGACCCCTTCCACCACC
WDR33_genomic_F	AGGCACACCTCTCAGTACGCTG
WDR33_genomic_R	CTAGTTTTCTGTCAATTCTTGCG

Supplemental Table S2: Plasmids used in this study

Name	Assembly	Source
pACEBac-CPSF160	N/A	This study (Epoch Life Science)
pACEBac-WDR33	N/A	This study (Epoch Life Science)
pACEBac-WDR33_1-576-TEV-SII	Gibson	This study
pACEBac-hFip1$_{FL}$	N/A	This study (Epoch Life Science)
pACEBac-hFip1$_{iso4}$	Gibson	This study
pACEBac-CPSF30$_{iso2}$	N/A	This study (Epoch Life Science)
pACEBac-symplekin	N/A	This study (Epoch Life Science)
pACEBac-symplekin-TEV-SII	Gibson	This study
pACEBac-symplekin$_{\Delta NTD}$-TEV-SII	Gibson	This study
pACEBac-CPSF100	N/A	This study (Epoch Life Science)
pACEBac-CPSF73	N/A	This study (Epoch Life Science)
pACEBac-CPSF73$_{D75N\ H76A}$	Gibson	This study
pACEBac-CPSF73$_{NTD}$	Gibson	This study
pACEBac-CPSF73$_{CTD}$	Gibson	This study
pACEBac-CStF77	N/A	This study (Epoch Life Science)
pACEBac-CStF77-TEV-SII	Gibson	This study
pACEBac-CStF64	N/A	This study (Epoch Life Science)
pACEBac-CStF50	N/A	This study (Epoch Life Science)
pACEBac-Pcf11	N/A	This study (Epoch Life Science)
pACEBac-Pcf11_769-1555-TEV-SII	Gibson	This study
pACEBac-Clp1	N/A	This study (Epoch Life Science)
pACEBac-CFIm68	N/A	This study (Epoch Life Science)
pACEBac-CFIm25	N/A	This study (Epoch Life Science)
pACEBac-CFIm25-TEV-SII	Gibson	This study
pACEBac-PAP	N/A	This study (Epoch Life Science)
pACEBac-PAP-TEV-SII	Gibson	This study
pACEBac-SSU72	N/A	This study (Epoch Life Science)
pET28a-3C-His$_6$-SSU72	Restriction-ligation	This study
pACEBac-RBBP6	N/A	This study (Epoch Life Science)
pACEBac-RBBP6_1-335-TEV-SII	Gibson	This study
pACEBac-RBBP6$_{Y228G}$_1-335-TEV-SII	Gibson	This study
pACEBac-RBBP6$_{P195G}$_1-335-TEV-SII	Gibson	This study
pACEBac-RBBP6$_{D43K\ R74E}$_1-335-TEV-SII	Gibson	This study
pACEBac-RBBP6_1-142-TEV-SII	Gibson	This study
pBig1a-CPSF160/ WDR33_1-576-TEV-SII/ CPSF30$_{iso2}$/ hFip1$_{iso4}$	Gibson	This study
pBig1a-CPSF160/ WDR33_1-576-TEV-SII/ CPSF30$_{iso2}$/ hFip1$_{FL}$	Gibson	This study
pBig1b-CPSF100/ CPSF73/ symplekin-TEV-SII	Gibson	This study
pBig1b-CPSF100/ CPSF73$_{D75N\ H76A}$/ symplekin-TEV-SII	Gibson	This study
pBig1b-CPSF100/ CPSF73/ symplekin$_{\Delta NTD}$-TEV-SII	Gibson	This study
pBig1b-CStF77-TEV-SII/ CStF64/ CStF50	Gibson	This study

pBig1b- Pcf11_769-1555-TEV-SII/ Clp1	Gibson	This study
pBig1a-CFIm25-TEV-SII/ CFIm68	Gibson	This study
pUCIDT-MS2-L3	Restriction-ligation	This study
Human_WDR33(C)-HTBH_repair	Gibson	Steven West (University of Exeter); modified in this study
Human_WDR33(C)_gRNA	N/A	Steven West (University of Exeter)

Supplemental Table S3: RNAs used in this study

**Name**	**Sequence**
5′-FAM-L3	AUGAUCUAGGAGACACAAUAAAGGCAAUGUUUUUAUUUGUA
MS2-L3	GGGGUCUAGACCUCGAGAAGCUUCGUACACCAUCAGGGUACGAGCUUGCCCUUGGCGU<br>ACACCAUCAGGGUACGACUAGUAUAUCUCGUACACCAUCAGGGUACGGAAUUCGGUA<br>CCCAACUCCAUGCUUAACAGUCCCCAGGUACAGCCCACCCUGCGUCGCAACCAGGAAC<br>AGCUCUACAGCUUCCUGGAGCGUCACUCGCCCUACUUCCGCAGCCACAGUGCGCAGAU<br>UAGGAGCGCCACUUCUUUUUGUCACUUGAAAAACAUGUAAAAAUAAUGUACUAGGAG<br>ACACUUUCAAUAAAGGCAAAUGUUUUUAUUUGUACACUCUCGGGUGAUUAUUUACCC<br>CCCACCCUUGCCGUCUGCGCCGUUUAAAAAUCAAAGGGGUUCUGCCGCGCAUCGCUAU<br>GCGCCACUGGCAGGGACACGUUGCGAUACUGGUGUUUAGUGCUCCACUUAAACUCAG<br>GCACAACCAUCCGCGGCAGCUCGGUGAAGUUUUCACUCCACAGGCUGCGCACCAUGAC<br>GG
$SV40_{AAUAAA}$	GGGAGACAUGAUAAGAUACAUUGAUGAGUUUGGACAAACCACAACUAGAAUGCAGUG<br>AAAAAAAUGCUUUAUUUGUGAAAUUUGUGAUGCUAUUGCUUUAUUUGUAACCAUUAU<br>AAGCUGC**AAUAAA**CAAGUUAACAACAACAAUUGCAUUCAUUUUAUGUUUCAGGUUCA<br>GGGGGAGGUGUGGGAGGUUUUUUAAAGCAAGUAAAACCUCGACGGAU
$SV40_{AACAAA}$	GGGAGACAUGAUAAGAUACAUUGAUGAGUUUGGACAAACCACAACUAGAAUGCAGUG<br>AAAAAAAUGCUUUAUUUGUGAAAUUUGUGAUGCUAUUGCUUUAUUUGUAACCAUUAU<br>AAGCUGC**AACAAA**CAAGUUAACAACAACAAUUGCAUUCAUUUUAUGUUUCAGGUUCA<br>GGGGGAGGUGUGGGAGGUUUUUUAAAGCAAGUAAAACCUCGACGGAU
